# The Role of Macrophage Migration Inhibitory Factor in Anesthetic-Induced Myocardial Preconditioning

**DOI:** 10.1371/journal.pone.0092827

**Published:** 2014-03-25

**Authors:** Andreas Goetzenich, Sandra Kraemer, Rolf Rossaint, Christian Bleilevens, Florian Dollo, Laura Siry, Setareh Rajabi-Alampour, Christian Beckers, Josefin Soppert, Hongqi Lue, Steffen Rex, Jürgen Bernhagen, Christian Stoppe

**Affiliations:** 1 Department of Thoracic, Cardiac and Vascular Surgery, University Hospital, RWTH Aachen, Aachen, Germany; 2 Institute of Biochemistry and Molecular Cell Biology, RWTH Aachen University, Aachen, Germany; 3 Department of Anesthesiology, University Hospital of the RWTH Aachen, Aachen, Germany; 4 Department of Anesthesiology and Cardiovascular Science, University Hospitals Gasthuisberg, KU Leuven, Leuven, Belgium; Kaohsiung Chang Gung Memorial Hospital, Taiwan

## Abstract

**Introduction:**

Anesthetic-induced preconditioning (AIP) is known to elicit cardioprotective effects that are mediated at least in part by activation of the kinases AMPK and PKCε as well as by inhibition of JNK. Recent data demonstrated that the pleiotropic cytokine macrophage migration inhibitory factor (MIF) provides cardioprotection through activation and/or inhibition of kinases that are also known to mediate effects of AIP. Therefore, we hypothesized that MIF could play a key role in the AIP response.

**Methods:**

Cardiomyocytes were isolated from rats and subjected to isoflurane preconditioning (4 h; 1.5 vol. %). Subsequently, MIF secretion and alterations in the activation levels of protective kinases were compared to a control group that was exposed to ambient air conditions. MIF secretion was quantified by ELISA and AIP-induced activation of protein kinases was assessed by Western blotting of cardiomyocyte lysates after isoflurane treatment.

**Results:**

In cardiomyocytes, preconditioning with isoflurane resulted in a significantly elevated secretion of MIF that followed a biphasic behavior (30 min vs. baseline: p = 0.020; 24 h vs. baseline p = 0.000). Moreover, quantitative polymerase chain reaction demonstrated a significant increase in MIF mRNA expression 8 h after AIP. Of note, activation of AMPK and PKCε coincided with the observed peaks in MIF secretion and differed significantly from baseline.

**Conclusions:**

These results suggest that the pleiotropic mediator MIF is involved in anesthetic-induced preconditioning of cardiomyocytes through stimulation of the protective kinases AMPK and PKCε.

## Introduction

Preconditioning of the myocardium is known to elicit an overwhelming release of mediators that activate various cytoprotective pathways and thus confer protection against the deleterious sequelae of events in ischemia and reperfusion (I/R). This phenomenon typically consists of two distinct phases: the early phase which starts immediately after the termination of a preconditioning stimulus and which protects the myocardium for 2–3 h, and a late protection period occurring after 12–24 h, which lasts for 2–3 days. The effect of preconditioning was originally demonstrated after single or multiple brief episodes of sub-lethal myocardial injury (e.g. hypoxia) [1], [2] and was subsequently extensively investigated. Of note, it was demonstrated to occur as well after application of volatile anesthetics [3]–[5]. However, despite various studies addressing the signal transduction cascade involved in anesthetic-induced pre–conditioning, the underlying mechanisms are still only partly understood. To date, the involvement of the pro-survival kinases protein kinase C (PKC) and adenosine monophosphate-activated protein kinase (AMPK) have been identified to play a pivotal role in anesthetic-induced cardioprotection [3]. In addition, further downstream signaling modules have been appreciated. For example, inhibition of the opening of mitochondrial permeability pores (mPTPs) has been found to play a pivotal role in preconditioning-induced cardioprotection [3], [6].

Recently, the pleiotropic cytokine macrophage migration inhibitory factor (MIF) has emerged as a promising mediator providing cardioprotection during myocardial ischemia and reperfusion [7]–[11]. MIF is an evolutionarily conserved protein that is known to act as a circulating mediator of the innate and acquired immune and inflammatory response and, when dysregulated, to contribute to a number of acute and chronic inflammatory disease conditions [12]. On the other hand, owing to its intrinsic redox activity and abundant cytosolic localization in several cell types, MIF can function as a potent antioxidant [11], [13], [14]. Overall, MIF has been demonstrated to provide significant cardioprotection through activation of the CD74/AMP kinase pathway in cardiomyocytes, through inhibition of c-Jun N-terminal kinase (JNK)-mediated apoptosis [7], [8] and through its antioxidant capacity [10]. Since MIF is known to be rapidly released in response to various stimuli and to share many characteristics that overlap with the mechanisms of anesthetic-induced preconditioning, both at the cellular and molecular level, we hypothesized that MIF plays a role in the anesthetic-induced preconditioning. Applying a cell system of primary rat cardiomyocytes, we expected MIF to be released from these cells after anesthetic-induced preconditioning and speculated that its cardioprotective effects might correlate with those mechanisms typically observed in anesthetic-induced preconditioning.

## Materials and Methods

All experiments were performed in compliance with the local institution's Ethical Review Committee and were approved by an animal protection representative at the Institute of Animal Research of the RWTH Aachen University Hospital in accordance with German Animal Protection Law §4, Section 3. All experimental procedures were approved by the Animal Care and Use Committee of the local authorities (AZ 50.203.2 AC, LANUV NRW, Germany).

### Proteins and reagents

Biologically active recombinant murine MIF (rMIF) was expressed and purified from the pET11b/BL21-DE3 expression, as described previously [15]. The endotoxin content of rMIF was lower than 5 pg LPS/μg as controlled by the Limulus QCL-1000 kit (Cambrex BioScience, Verviers, Belgium). All other reagents were obtained from Sigma (St. Louis, MO, USA), Merck (Darmstadt, Germany) or Roth (Karlsruhe, Germany), if not stated otherwise.

### Culture of rat ventricular myocytes

Cardiac ventricular myocytes were isolated from hearts of 2–4 day-old Wistar rats by trypsin digestion as described previously [16]. The cell characteristics of cardiomyocytes equals to an adult phenotype, which has repeatedly been evaluated before [16]. Briefly, rats were decapitated and the ventricles were collected in ice-cold CBFHH Medium (calcium- and bicarbonate free Hanks buffer containing 20 mM HEPES, pH 7.4, 140 mM NaCl, 5.4 mM KCl, 0.81 mM MgSO_4_, 0.44 mM KH_2_PO_4_, 0.34 mM Na_2_HPO_4_ and 5.6 mM glucose). After the tissue was minced into pieces smaller than 1 mm, 0.25% trypsin (1∶250, Difco, BD, Franklin Lakes, NJ, USA) was added to the suspension. After 10 min incubation the supernatant was discarded. Trypsin digestion then occurred in several cycles of trypsination and inactivation with fetal calf serum (FCS) and Dnase (DNAse1 bovine pancreas, Sigma-Aldrich) to eliminate free DNA until the tissue was completely digested. Supernatants containing isolated cells were collected and the cells were plated on 6-well dishes coated with 5 mg/ml fibronectin in 0.02% gelatin at a density of 150.000 cells/mm^2^. Cardiomyocytes were cultured for 12 days in DMEM supplemented with 10% horse serum, 2% CEE chicken embryo extract, 2 mM L-glutamine and 100 U/ml penicillin/streptomycin to develop an adult phenotype [16]. After creating a homogenous two-dimensional layer and showing a homogenous contraction profile, cells were randomized to the different treatment groups.

### Immunofluorescence

For analyzing MIF receptor expression via confocal fluorescence microscopy, 300.000 freshly isolated cardiomyocytes were previously counted with a hemocytometer and subsequently seeded into μ-dishes (35 mm high, Ibidi, Munich, Germany) and cultivated for 12 days. Cells were then fixed with 3.6% paraformaldehyde (supplemented with 0.1% Hoechst 33342 for nuclear staining) for 20 min and subsequently permeabilized with 0.3% Triton X-100 and 5% FCS in PBS for 1 h. Antibodies against murine CD74 (C16, sc-5438, Santa Cruz Biotechnology, Heidelberg, Germany), CXCR2 (bs1629R, Bioss, Woburn, USA), and CXCR4 (ab2074, Abcam, Cambridge, USA) or appropriate isotype controls goat IgG (ab37373, Abcam, Cambridge, USA) or rabbit IgG (sc-2027, Santa Cruz Biotechnology, Heidelberg, Germany) were diluted 1∶200 in PBS (containing 0.3% Triton X-100 and 2% BSA) and cardiomyocytes were incubated for 2 h at room temperature. Following 3 washing steps with PBS fluorescently labeled secondary antibodies (Alexa Fluor 633 goat anti-rabbit IgG, Alexa Fluor 633 donkey anti-goat IgG Invitrogen, Darmstadt, Germany) were also diluted 1∶200 in PBS (containing 0.3% Triton X-100 and 2% BSA) and incubated with the cardiomyocytes for 1 h at room temperature. Stained cardiomyocytes were analyzed by laser scanning fluorescence microscopy using an LSM710 confocal microscope (Zeiss, Jena, Germany).

### Flow cytometry

Cardiomyocytes were detached with Accutase (PAA, Piscataway, USA) before being subjected to flow cytometry. For each measurement 300.000 cells were washed once with ice-cold FACS buffer (PBS, pH 7.2, containing 0.5% bovine serum albumin and 0.1% sodium azide). Cells were then labeled with anti-CD74-FITC (BD Bioscience, Heidelberg, Germany) or the appropriate isotype control in 25 μl FACS buffer for 30 min on ice. After washing with FACS buffer cells were analyzes on a FACS Canto II (BD Bioscience, Heidelberg, Germany).

### Preconditioning with isoflurane

For preconditioning of cardiomyocytes, volatile isoflurane treatments were performed in a self-designed heated incubation chamber (WALLA GmbH, Würselen, Germany) connected to a multichannel flow regulator (Bronkhorst-Mättig, Kamen, Germany) and an isoflurane vapor (Eickemeyer, Tuttlingen, Germany). Oxygen, carbon dioxide and isoflurane levels were continuously monitored on a Datex Ohmeda multichannel patient monitor (GE Healthcare, Munich, Germany). Cardiomyocytes were treated for 4 h with 1.5% isoflurane (concentration in the medium), which is commonly used in our own institutional practice. Control cells (sham group) were treated in the same chamber without isoflurane. *A medium exchange was performed after the preconditioning treatment,* cells were cultured under normal conditions for up to 48 h and samples taken at different time points. Cell-free supernatants were taken for detection of MIF secretion and replaced by an equal amount of medium taken from a separate well that had been carried along through the treatment. Cells were directly lysed in 200 μl Lämmli sample buffer, sonified, boiled and subjected to SDS-PAGE and Western blot.

### Stimulation with recombinant MIF

For direct stimulation experiments, adult rat cardiomyocytes were incubated with 100 ng/ml of recombinant murine MIF for different time points. Cells were then lysed in 200 μl Lämmli sample buffer, sonified, boiled, and subjected to SDS-PAGE and western blot.

### Cell viability after terminal hypoxia

The cell viability was assessed by two different methods. The Promokine Apoptotic/Necrotic/Healthy kit (PromoCell GmbH, Heidelberg, Germany; data not shown) was used for qualitative visualization of necrotic and apoptotic cells, whereas Trypan blue staining was applied for quantitative cell counting.

### MIF measurement

To assess intracellular MIF concentrations in rat cardiomyocytes, cells were lysed using the freeze-thaw method. Cells were detached via trypsination and re-suspended in PBS at a concentration of 150.000 cells/ml. Suspensions were then frozen in liquid nitrogen, thawed at 37°C and subjected to sonication. Cell debris was removed by centrifugation at 16.000×g for 10 min. Freeze-thaw cycles were repeated 3 times to ensure complete cell lysis.

MIF levels in cell culture supernatants and lysates were assessed using a mouse/human combination ELISA technique that represented a modification of a described procedure [17]. Briefly, 96 well Maxisorp Immuno Plates (Nunc, Rochester, USA) were coated with 1.5 μg/ml anti-mouse MIF mAB XIV.14.3 (kind gift of Prof. Bucala, Yale) overnight, washed and blocked for 1 h with PBS containing 1% BSA and 5% sucrose. 100 μl of lysates, cell-free supernatants or serial murine MIF dilutions (in either PBS or cardiomyocyte medium respectively) were added to each well and incubated for 2 h. After washing, the wells were incubated with the biotinylated anti-human MIF mAB BAF289 (0.2 μg/ml in TBS, containing 0.1% BSA and 0.05% Tween20) for 2 h. Washed wells were then incubated with peroxidase-conjugated streptavidin (Roche Applied Sciences, Mannheim, Germany) for 20 min. After addition of TMB substrate solution (Pierce, Rockford USA), reaction was stopped with 0.5 N H_2_SO_4_ and immune complexes were quantified in a standard microtiter plate reader at 405 nm.

### Western blotting

For Western blotting primary cardiomyocytes were directly lysed in 200 μl Lämmli buffer per well of a 6-well plate. Lysates were sonified and boiled at 95°C for 5 min before being subjected to SDS-Page and Western blotting, For protein detection, membranes were probed with antibodies against phospho-AMPKα (Thr172, dilution 1∶1000, Cell Signaling Technologies), phospho-SAPK/JNK (Thr183/Tyr185, clone 81E11, dilution 1∶1000, Cell Signaling Technologies), phospho-PKCε (Ser729, dilution 1∶1000, Santa Cruz) and Actin (clone C4, MP biomedicals). Blots were developed with the Super Signal West Dura Extended Duration ECL reagent (Pierce/KMF Laborchemie, St. Augustin, Germany), using an appropriate peroxidase-conjugated secondary antibody. Chemiluminescence was detected with the LAS-3000 imager equipped with a CCD camera with a 16 bit resolution and band intensities were quantified using the AIDA image analyzer software (Fuji/Raytest Isotopenmessgerät GmbH). Actin was used as a loading control and all bands were normalized to it.

### Quantitative real time polymerase chain reaction

Eight hours after isoflurane treatment, total RNA was isolated using a commercially available RNA/Protein extraction kit (NucleoSpin RNA/Protein, Machery-Nagel, Düren, Germany) and reverse-transcribed into cDNA using a high-capacity reverse transcription kit (Applied Biosys-temsW, Carlsbad, CA, USA). The PCR reaction was performed using 50 ng of cDNA (TaqMan universal PCRmix, Applied Biosystems) and specific TaqMan probes for MIF (Rn00821234_g1) and the housekeeping gene hypoxanthin-guanine phosphoribosyltransferase (HPRT, Rn01527840_m1) on a StepOne Plus Cycler (Applied Biosystems). The relative quantity (RQ) values were calculated according to the ΔΔCt method, which reflects the differences in the threshold for each target gene relative to HPRT and the sham-operated rat.

### Statistical analysis

All data were statistically analyzed using a commercially available software package (SPSS 19.0 (SPSS inc., Chicago, IL, USA).

All data were tested for normal distribution using the Shapiro-Wilk-W-test. Normally distributed results of single measurements were compared between the groups using the student's t-test. A repeated measurement analysis of variance was applied where appropriate. Given the explorative character of our study, the significance level of the results was not adjusted for multiple hypotheses (i.e., for all markers of Western Blotting in this investigation). Non-parametric data were compared using the Wilcoxon signed rank test. In case of significant results, post-hoc testing was performed using the Bonferroni test and (for not normally distributed data) the Wilcoxon signed rank test with the Bonferroni adjustment for multiple measurements, respectively. In all cases, a level of P<0.05 was considered statistically significant.

## Results

### Experimental set-up

**Figure 1:**
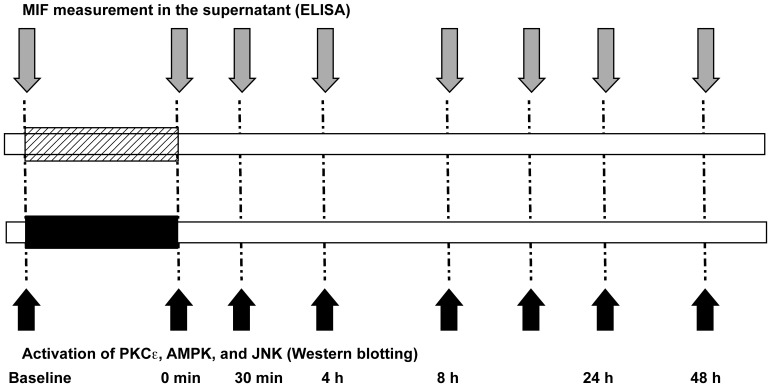
Protocol for experimental studies. All isolated cardiomyocytes were subjected to either preconditioning stimulus of isoflurane or room air for 4

The experimental set-up and treatment schemes applied are summarized in Figure 1. The isolated cardiomyocytes were standardized subjected to either preconditioning stimulus of room air or isoflurane for 4 h. Subsequently cells were harvested and lysed for detection of phosphorylated kinases by western blot. Simultaneously the supernatants from cardiomyocytes were taken and stored until the latter measurement of secreted MIF.

### Characterization of rat cardiomyocytes: MIF expression and MIF receptors

First, we aimed to determine the intracellular MIF concentration in the cardiomyocytes. Therefore, we quantitated MIF in freeze-thaw lysed resting cardiomyocytes (150.000 cells/ml). A concentration of 25.59±4.65 ng/ml, equaling to 0.16 pg/cell was obtained. For comparison, monocytes/macrophages exhibit MIF concentrations of about 0.15 pg/cell [12]. Thus, cardiomyocytes contain substantial concentrations of prestored intracellular MIF amenable to secretory stimuli.

**Figure 2:**
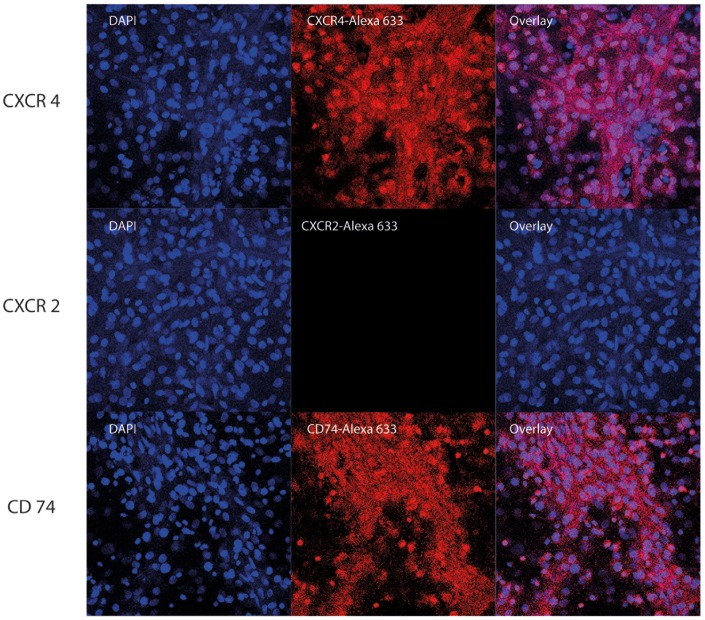
Characterization of MIF receptors by confocal microscopy. Rat cardiomyocytes were grown in an Ibidi μ-dish for 12 days. They were fixed with paraformaldehyde, permeabilised with Triton X-100 and stained with antibodies against CD74 (A) and CXCR4 (B) and fluorescently labeled secondary antibodies (Alexa 633). Nuclei were stained with Hoechst33342, a cell membrane permeable, DNA-binding fluorophore staining nuclei of cells with blue fluorescence.

To determine whether secreted MIF might exert an auto/paracrine effect on isolated cardiomyocytes, we next investigated whether the cardiomyocytes expressed the MIF-specific receptors CXCR2, CXCR4, or CD74 [18]. As flow cytometric measurements are inherently difficult to perform with primary rodent cardiomyocytes, we elected to first use confocal fluorescence microscopy. We were able to detect CXCR4 in the isolated rat cardiomyocytes but failed to measure detectable levels of CXCR2 (Figure 2, Figure S1). CD74 was detectable by confocal microscopy, but the obtained signals did not necessarily prove the existence of CD74 on the cell surface. We therefore additionally performed a flow cytometric analysis. This method confirmed the expression of CD74 on the cell surface of the isolated cardiomyocytes (Figure S2).

### Preconditioning with isoflurane

**Figure 3:**
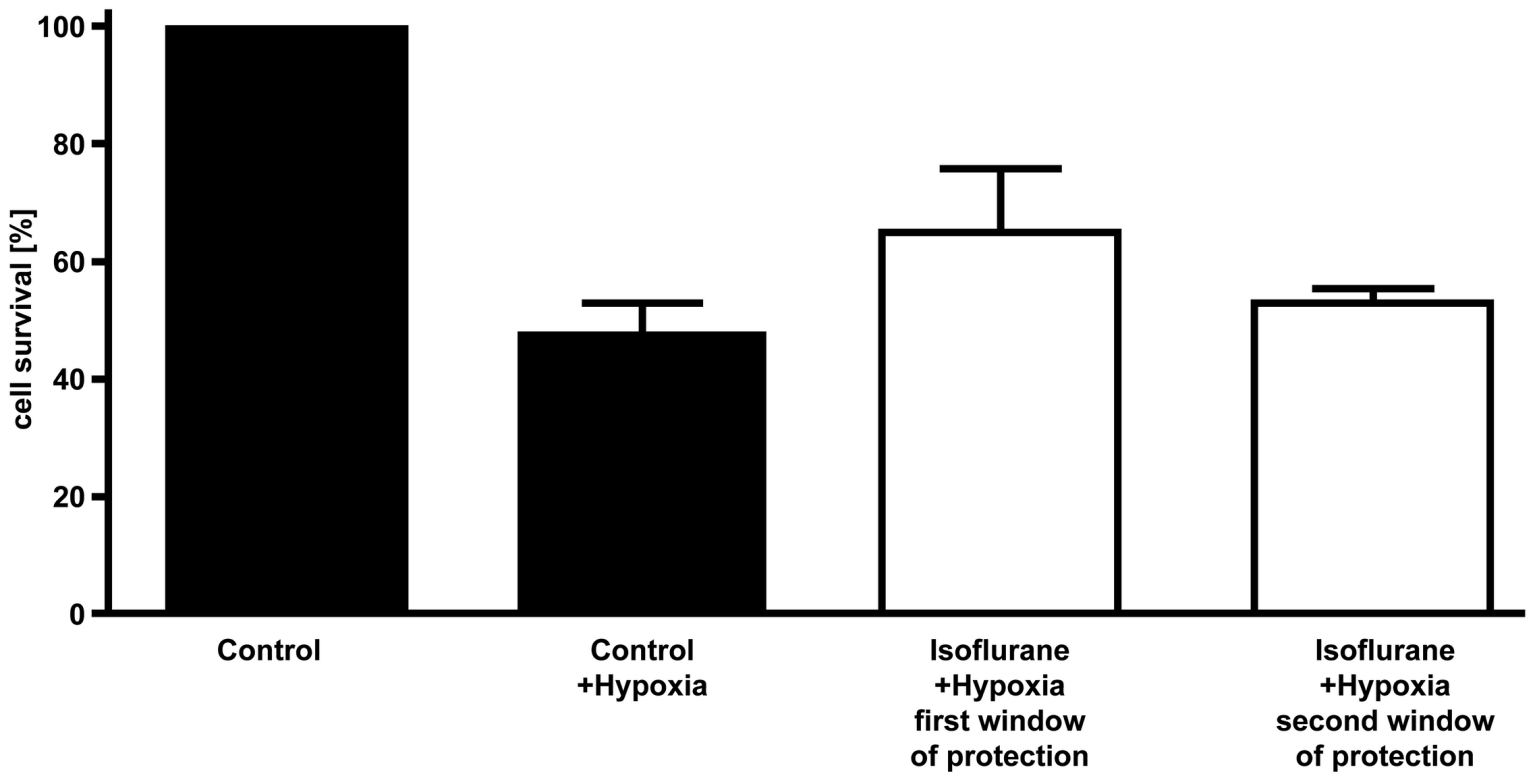
Measurement of survival. Cardiomyocytes from sham group and after preconditioning were subjected to hypoxia (5 hours) during the first and second window of protection in order to compare the survival between both groups and demonstrate the effect of preconditioning. Cell survival is given in percent of seeded cells. Whereas unpreconditioned cells were diminished to 48±2%, isoflurane preconditioned cells reached a survival ratio of 65±5% (n = 6 per group, p = 0.0061).

For anesthetic-induced preconditioning (AIP) experiments, cardiomyocytes isolated from 2–4 day-old rats were treated with 1.5% isoflurane for 4 h. The known protective effects of isoflurane preconditioning compared to control cells treated with air alone (sham group) on cell survival after 5 h of terminal hypoxia (<1%) were confirmed by a Trypan blue cell count (n = 6 per group). Cell viability was found to be increased by 17±5% (p = 0.0061) when cardiomyocytes were pretreated with isoflurane (Figure 3).

### MIF is secreted after preconditioning with isoflurane

**Figure 4:**
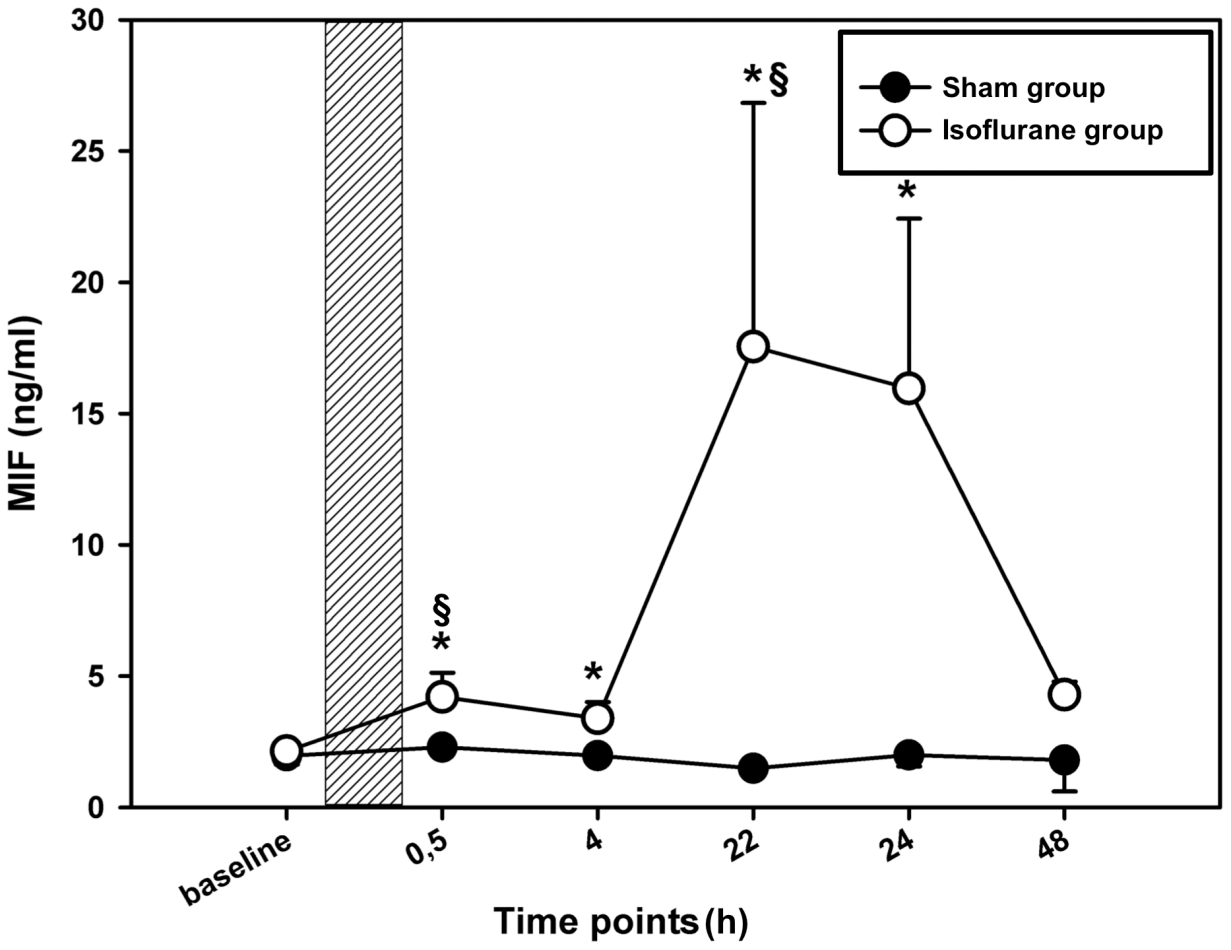
MIF secretion after anesthetic induced preconditioning. Comparison of MIF secretion in the supernatant of isolated cardiomyocytes after isoflurane or control treatment. 12 days after isolation cardiomyocytes were placed in an incubation chamber and treated for 4% isoflurane or normal room air as control (sham group). At the indicated time points, cell-free supernatants were collected and MIF concentrations were measured using a modified mouse/human combination-ELISA [17]. Data represented means ± SEM of at least 3 independent experiments. The shaded area indicates duration of preconditioning. *(**) = p<0.05(0.01) vs. baseline, §(§§) = p<0.05(0.01) vs. sham group.

To investigate whether preconditioning with isoflurane triggered MIF release from cardiomyocytes, the MIF concentration in the supernatants of the cardiomyocytes was measured after preconditioning with isoflurane at predefined time points that are known to be relevant in anesthetic-induced preconditioning [1], [2]. To investigate the autocrine/paracrine role of MIF in anesthetic-induced preconditioning, supernatants and cell lysates were taken at different time points before and after isoflurane treatment as indicated in Figure 1. Immediately after termination of preconditioning, MIF levels showed a slight increase over baseline values. Thirty minutes after termination of the application MIF levels were significantly increased. In addition, we observed a second significant rise of MIF secretion 22–24 h after preconditioning as compared to the initial baseline value (22 h: 17.5±12 vs. 2.1±0.3 ng/ml; p = 0.039 and 24 h: 15.9±10.9 vs. 2.1±0.3 ng/ml; p = 0.026). Measured MIF levels further differed significantly from MIF levels that were measured in a sham group of cardiomyocytes (24 h: 15.9±10.9 vs. 1.03±0.8 ng/ml; p = 0.041) (Figure 4). In conclusion, the secretion analysis revealed a biphasic MIF release response after isoflurane-induced preconditioning.

**Figure 5:**
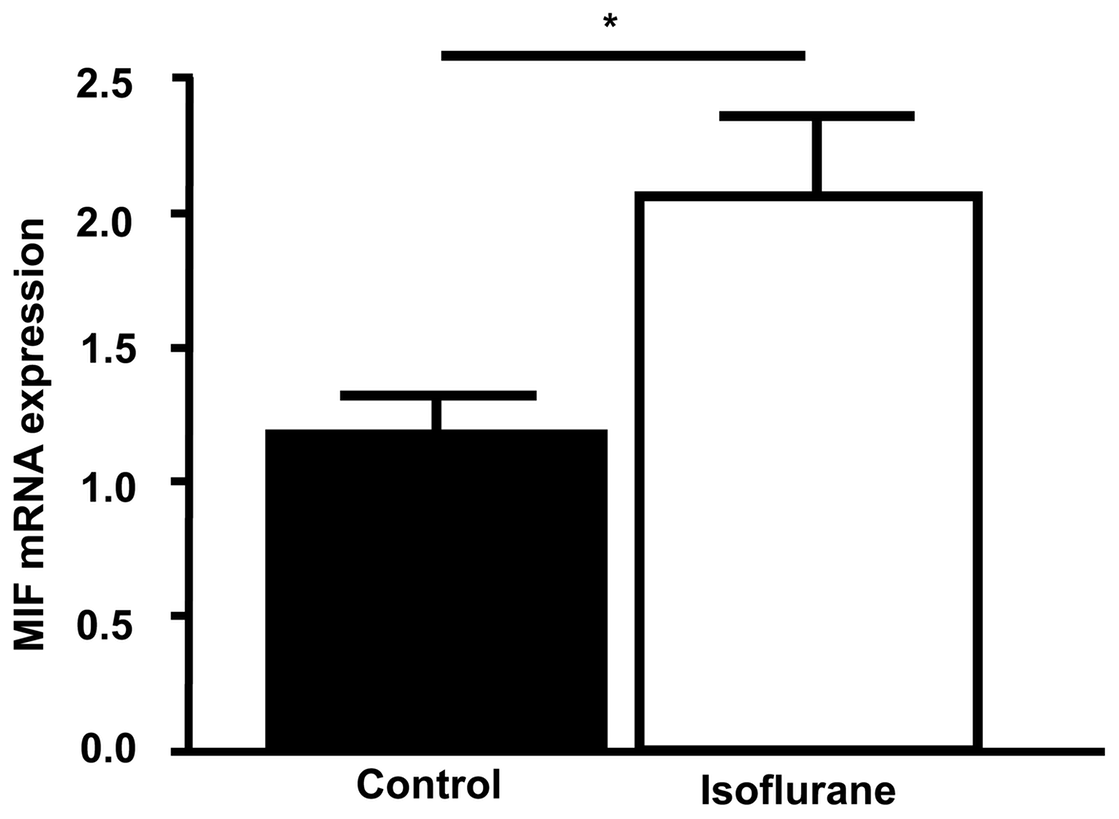
Isoflurane preconditioning stimulates MIF mRNA expression in cardiomyocytes. Cardiomyocytes were treated with isoflurane (4 hours; 1.5%) for preconditioning and consecutively compared to cardiomyocytes that were exhibited to room air in the incubation chamber. 8 hours after the treatment, mRNA was isolated and qPCR was performed. mRNA levels were normalized to GAPDH. Data represented means ± SEM of at least 3 independent experiments. *(**)  =  p<0.05 (0.01) vs. sham group.

Considering this biphasic secretion profile with its major peak at 22–24 h, we surmised that part of the response could be based on MIF de-novo synthesis. Messenger RNA was isolated 8 h after preconditioning (assuming that an mRNA peak would occur several hours before the observed secreted protein peak) and qPCR performed for quantification. In comparison to the untreated sham group, a roughly twofold increase in MIF mRNA levels (isoflurane vs. control: p = 0.030) (Figure 5) was observed, which could at least partly account for the 22–24 h MIF secretion peak.

### Preconditioning with isoflurane activates AMPK and PKCε but shows an ambivalent effect on JNK1/2

To begin to test for an association between the observed MIF secretion effect and an activation effect on known cardioprotective kinases, we simultaneously determined the phosphorylation status of AMPK, PKCε and JNK1/2 at various time points after preconditioning (see Figure 1).

#### PKCε

**Figure 6:**
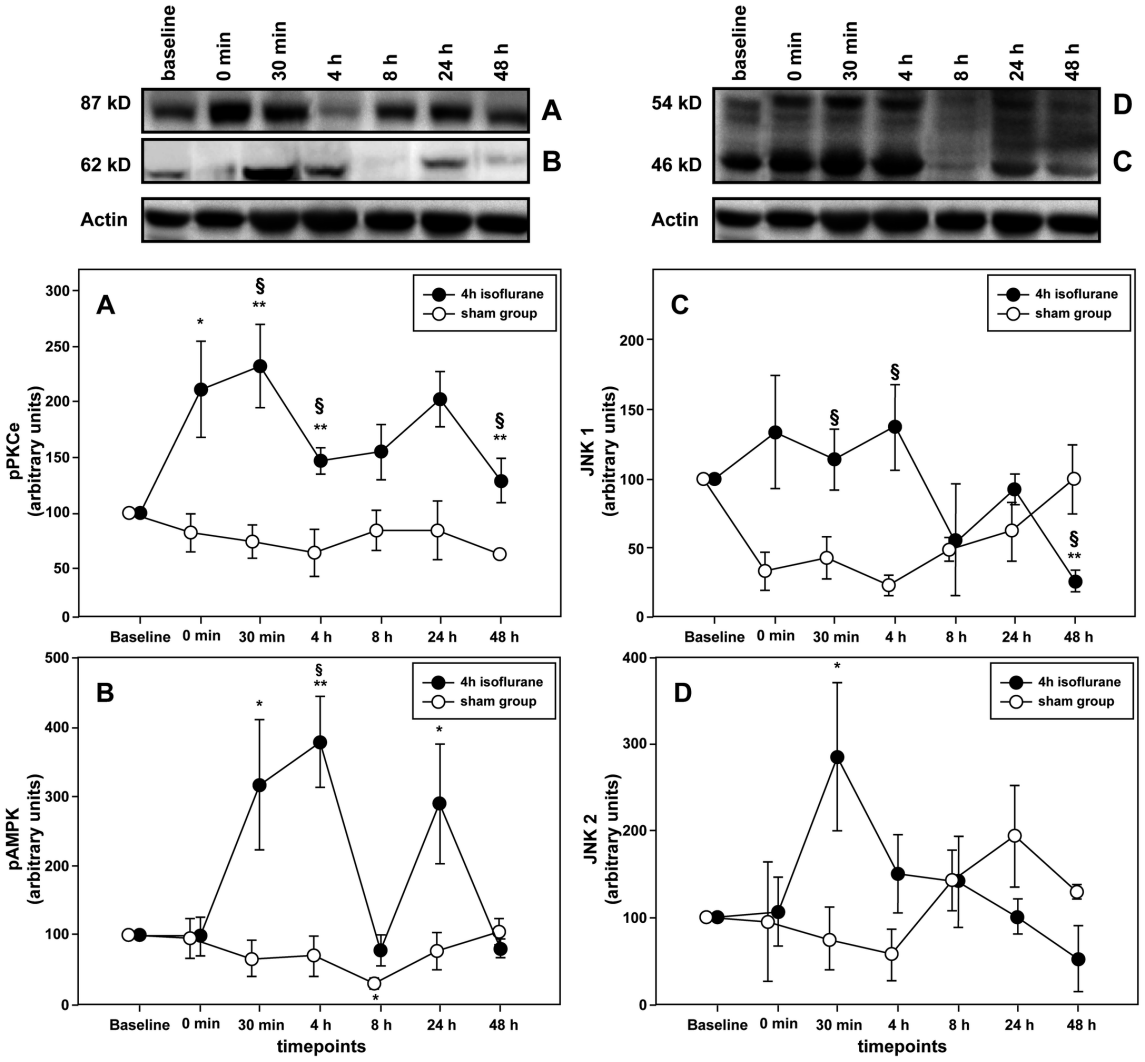
Increased MIF levels are associated with activation of protective kinases PKCε and AMPK. Relative activation of protein kinases was assessed at the indicated time points after preconditioning with 1.5% isoflurane for 4 h by western blotting. Band intensities were normalized to actin. Data shows a biphasic activation for PKCε (A) and AMPK (B) that conforms with the elevated MIF levels after preconditioning. No effect was measured for the activation of either JNK1 (C) or JNK2 (D). Data represented means ± SEM of at least 3 independent experiments. *(**) = p<0.05(0.01) vs. baseline; §(§§) = p<0.05(0.01) vs. sham group

Since PKCε has repeatedly been shown to convey preconditioning effects after AIP, we first analyzed the levels of phosphorylated PKCε. PKCε values upon isoflurane preconditioning were found to substantially and rapidly increase and remained elevated over the entire time course, when compared to baseline and the sham group. Of note, the analysis also revealed a pronounced biphasic activation behavior during the observation period with a maximum reached approximately 30 min after termination of preconditioning (baseline vs. 30 min: p = 0.021; sham group vs. 30 min: p = 0.021). A second peak was seen after 24 h (p = 0.048) (Figure 6A, Figure S3A).

#### AMPK

Recent studies demonstrated that cardioprotection by MIF is based on a MIF-triggered activation of the CD74/AMP kinase pathway in cardiomyocytes [7]. Likewise, AIP is supposed to be mediated by reactive oxygen-induced activation of 5′AMP-activated protein kinases [19]. We observed a significant elevation in the activation of AMPK throughout the observation period (p = 0.000). The phosphorylation of AMPK levels differed significantly 30 min (30 min vs. baseline: p = 0.020) and 24 hours after termination of preconditioning (24 h vs. baseline: p = 0.047), whereas maximal activation of AMPK was observed 4 hours after preconditioning (4 h vs. baseline: p = 0.004). Comparison with the sham group revealed a significant difference between the groups 4 h after activation (ISO vs. Sham: p = 0.013) (Figure 6B, Figure S3B). Since the effect of MIF on JNK1/2 activation is complex and its role in preconditioning remains largely unknown, we analyzed the effect of MIF on the isoenzymes JNK1 and JNK2 phosphorylation separately.

#### JNK1

The activation of JNK1 showed visible changes over time, which however did not reach statistical significance (p = 0.120). Nevertheless, a reduction of JNK1 activity about 8 h after preconditioning was observed and reached a minimum after 48 h (48 h vs. baseline: p = 0.000). When compared to the sham group, activation was significantly higher in the treatment group either 30 min (sham group vs. 30 min: p = 0.038) or 4 h after preconditioning (sham group vs. 4 h: p = 0.012) (Figure 6C, Figure S3C).

#### JNK2

With the exception of the 30 min time point that showed maximal activation of JNK2 (30 min vs. baseline: p = 0.049), phosphorylation decreased within the observation period but did not show any significant change. No difference could be detected in JNK2 activation pattern between the sham and isoflurane group (Figure 6D, Figure S3C).

### MIF directly activates cardioprotective kinases

**Figure 7:**
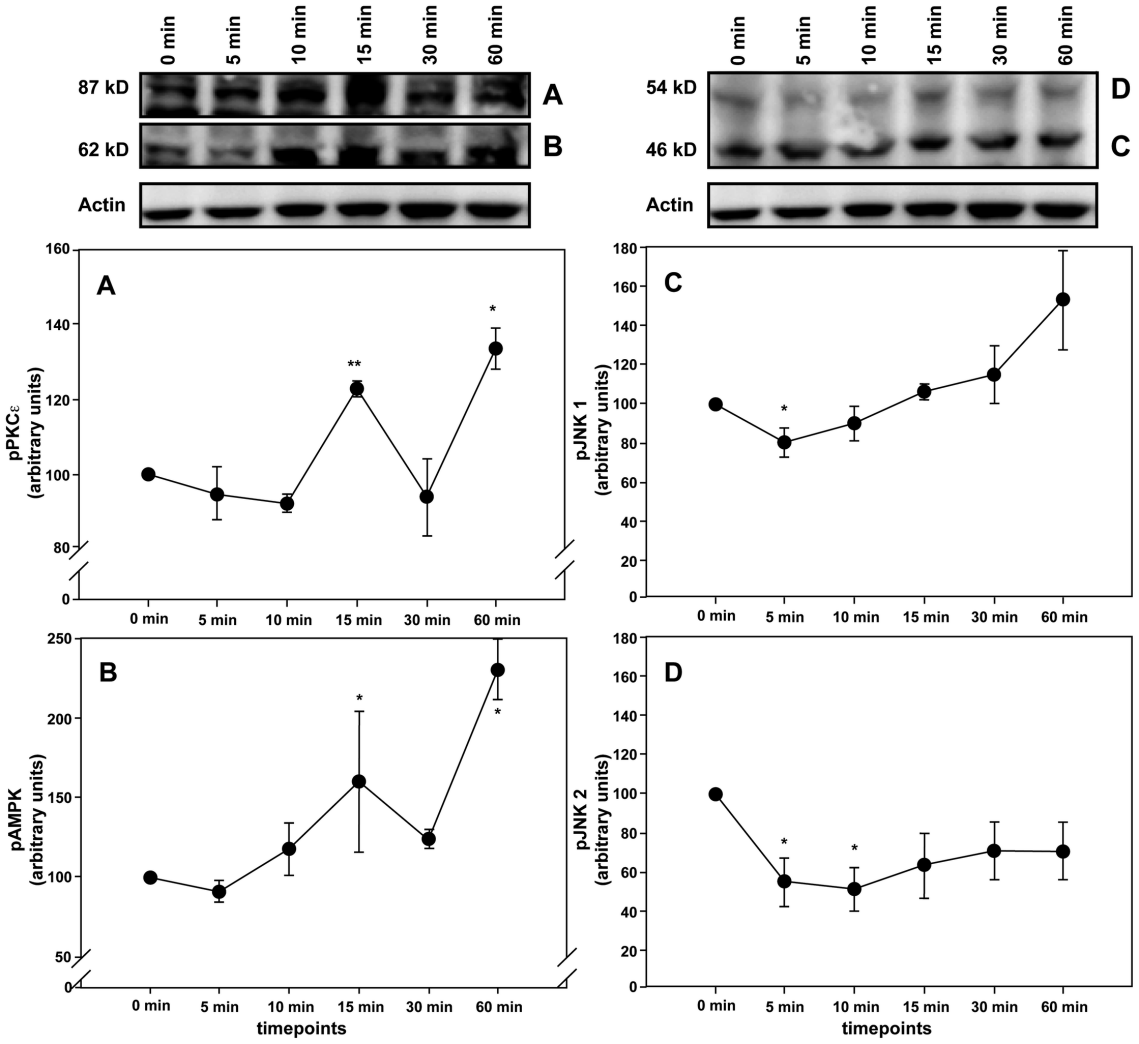
Recombinant MIF activates AMPK and PKCε whereas it inhibits phosphorylation of JNK1/2. Cardiomyocytes were incubated with 100/ml recombinant mouseMIF for the indicated time points. Relative activation PKCε (A) AMPK (B), JNK1 (C) and JNK2 (D) was assessed via western blotting. Band intensities were normalized to actin. Data represented means ± SEM of at least 3 independent experiments. *(**)  =  p<0.05 (0.01) vs. baseline.

Since MIF has repeatedly been shown to induce the activation of the protective kinase AMPK while inhibiting JNK [7], [8], we investigated a potential association between the observed MIF secretion and kinase activation (or deactivation, respectively) patterns after preconditioning with isoflurane. Moreover, to study whether AIP-induced secreted MIF might functionally contribute to the activation of the protective kinases, we asked if recombinant MIF was capable of activating the kinases in isolated rat cardiomyocytes. Application of exogenous mouse MIF resulted in a significant increase of PKCε and AMPK phosphorylation after 15 and 60 min (Figure 7A–B). In contrast, the activity of JNK1 and JNK2 was initially high and then reduced (Figures 7C–D). This observation suggested a causal effect of MIF on isoflurane-triggered signaling responses.

Autocrine/paracrine effects of secreted MIF after AIP could therefore indeed be responsible for the observed activation of AMPK and PKCε as well as for the inhibition of JNK1/2.

## Discussion

To our knowledge, the data of the present study are the first indicating a role of MIF in anesthetic-induced preconditioning of cardiomyocytes. A biphasic MIF secretion profile was noted immediately after termination of preconditioning as well as 24 h thereafter. Of note, this coincides with the two known time windows of early and late phase preconditioning. We also observed an activation of the cardioprotective kinases AMPK and PKCε, which coincided with the increase in MIF values in the supernatants of preconditioned cardiomyocytes. Furthermore, we confirmed that recombinant MIF directly triggers phosphorylation of these two kinases in isolated cardiomyocytes.

Coronary artery disease is the leading cause of death worldwide according to the World Health Organization. Despite substantial technical advances in coronary revascularization, the ageing population with increasing prevalence of co-morbidities poses a serious challenge that has stimulated high research activity about myocardial preservation strategies [20]. In particular high-risk patients, including those with increasing age or combined surgical procedures, are most susceptible to perioperative myocardial injury and infarction. For these patients, anesthetic preconditioning of the myocardium belongs to the novel therapies that are supposed to stimulate intracellular signal transduction pathways, which confer cardioprotection [21], [22]. Although Gao and colleagues previously reported a deleterious effect of MIF during prolonged myocardial reperfusion [23], emerging evidence indicates the MIF induced cardioprotective effects [7], [8], [24], [25], which are comparable to those, which are known to be induced by anesthetic-preconditioning [19], [26], [27]. Indeed, our data demonstrated a biphasic MIF secretion curve that was stimulated by application of isoflurane. While the first MIF peak at 30 min was significant but marginal, the secretion peak at 24 h represented strong and maximal MIF release, which together might point to a role for MIF in both the early and late phase of preconditioning. Crystall et al. suggested that an enhanced antioxidant capacity would be at least in part responsible for the protective effects that result from isoflurane-induced preconditioning during the late phase (second window) of preconditioning [28]. MIF itself is capable of attenuating oxidative stress owing to its intrinsic thiolprotein oxidoreductase activity (TPOR) [9], [11], [13]. The MIF sequence contains three Cys residues, which have been identified to contribute to several of the functional properties of MIF. Cys-57 and Cys-60 form a CXXC motif that has catalytic TPOR activity and is a distinct hallmark of the TPOR family of proteins [9], [10]. Thus, the later increase in MIF should serve to enhance the antioxidant capacity in the myocardium, a conclusion supported by the prior observation of Koga and Luedike [9], [14]. This supposed function in the “late” window could be of particular interest for patients with myocardial ischemia/reperfusion (e.g. cardiac surgery) since cardiac dysfunctions, such as stunning or atrial fibrillation, frequently occur after restoration of coronary blood flow and lasts for at least 1–2 days [11], [29].

Although MIF secretion and activation of protein kinases were only measured at distinct time points, the observed pattern complies with a biphasic secretion after preconditioning. At first, MIF might have been liberated from pre-formed pools in cardiomyocytes as a consequence of ischemia and reperfusion. The second MIF peak might result from induction of MIF mRNA de novo synthesis. Notably, the second increase of MIF content in the supernatant was seen simultaneously to the onset of the well-known second window of preconditioning [3], [30] and is consistent with previous findings that demonstrated a biphasic MIF secretion after application of hypoxia [31], [32]. This analogous behavior supports the hypothesis that MIF could have a significant causal role in the signal transduction pathways induced by preconditioning.

Over the past decade a number of studies have demonstrated that MIF regulates key functions during myocardial ischemia/reperfusion injury with an overall cardioprotective effect [7], [8], [11]. Considering the underlying mechanisms, this property involves activation of AMPK and inhibition of JNK. Therefore, we measured activation of theses kinases at the predefined time points of this study. Indeed, our results revealed a close link between preconditioning-induced MIF secretion and activation of AMPK, which was observed immediately after termination of preconditioning and 24 hours later. Furthermore, by application of recombinant MIF to isolated cells, we confirmed that MIF can cause activation of AMPK in cardiac myocytes, which supports the concept of a MIF-induced para-/autocrine activation of AMPK in cardiomyocytes. However, although striking this naturally does not exclude a major influence of other factors such as additional cytokines, adipokines, and other cell-protective mediators. Future studies are needed to systematically scrutinize the complexity and interplay of the underlying mechanisms. In contrast, we could not confirm an association between MIF secretion and decreased JNK activation as previously shown by Qi et al. in situations of protection against ischemia/reperfusion injury [8]. However, it still remains controversial discussed if AIP mediates inhibition of JNK-induced apoptosis. In the same vein, it has previously been shown that preconditioning-induced cardioprotection involves the family of mitogen-activated protein kinases (MAPK) p38 and ERK but not JNK, as the blockade of JNK during preconditioning had no effect on infarct size [33]. Therefore, the present findings have to be considered critically in view of the ongoing controversial discussion about the involvement of JNK within the anesthetic induced preconditioning. Furthermore we suppose that the induced effects of anesthetic-preconditioning are more complex and cannot be idealized by our stimulation experiments alone.

An impressive body of evidence considers the activation of PKC (mainly its ε-isoform) as a major signaling component of anesthetic-induced preconditioning [34]. A previous study has shown that a specific inhibition of PKCε abolishes the protective effects that are induced by preconditioning stimuli [35]. PKCε has been demonstrated to interact with various mitochondrial proteins including components believed to constitute the mitochondrial permeability transition pore [36]. The resulting effect was shown to inhibit the deleterious effects of Ca^2+^-induced mitochondrial swelling, which is commonly used as index of mitochondrial pore opening in in vitro models. Furthermore it is important to note that an activation of PKCε leads to a phosphorylation of kinases from the family of mitogen-activated protein kinases (MAPK), including p38, ERK and JNK [3]. The latter might be of particular interest when considering the obvious discrepancy between elevated MIF levels in the supernatants of cardiomyocytes and increase of JNK activation, which thus might have resulted from initial PKC activation. Given the obvious crucial role of PKCε, we as well investigated if PKCε activity in cardiomyocytes might be stimulated by exogenous MIF. Indeed, we observed a time-dependent activation of PKCε that further strengthens our hypothesis that MIF secretion from cardiomyocytes might lead to an auto/paracrine activation of PKCε. Data from previous studies are sparse and there is only one study indicating a close link between MIF and PKC. In fact, our study is the first one to identify PKCε as a target of MIF regulation. Interestingly, Takahashi et al. suggested MIF expression to result from activation of PKC [31]. These data are in line with our findings that showed an initial activation of PKCε with following increased MIF expression in comparison to the sham group. Future studies in whole animal models are needed to prove this important link between MIF and activation of PKCε.

We acknowledge that the present study suffers from several limitations. While an inclusion of more measuring points would have been desirable, we have only considered the MIF levels and activity of protein kinases at distinct time points. In addition we still cannot rule out, whether the activation of kinases results from various mediators others than MIF that could be secreted after preconditioning with isoflurane. In fact, it is likely that MIF is part of a cocktail of factors that contribute to the preconditioning effects, but our data suggests that it might be a critical component within preconditioning. On the other hand we were able to show a direct MIF-triggered activation of AMPK and PKCε. While high amounts of a MIF specific antibody (not commercially available) would have been needed for a sufficient inhibition, we were however unable to provide a further mechanistically investigation of the present observations. Furthermore we acknowledge that although volatile anesthetics share many beneficial characteristics and have repeatedly been shown to provide preconditioning effects by comparable mechanisms, present findings may vary between different anesthetics and should be systematically investigated in future.

## Conclusion

In summary, using a cellular cardiomyocyte model, we show that MIF is both correlatively and functionally involved in the protective effects observed in isoflurane-induced preconditioning. We have demonstrated a close association between MIF secretion and the activation of several cardioprotective kinases after application of isoflurane, which together with a direct effect on cardiomyocyte kinases, indicate a significant role of this pleiotropic cytokine in anesthetic-induced preconditioning.

## Supporting Information

Figure S1 **Control - characterization of MIF receptors by confocal microscopy.** Rat cardiomyocytes were grown in an Ibidi μ-dish for 12 days. They were fixed with paraformaldehyde, permeabilised with Triton X-100, treated with the appropriate isotype controls and fluorescently labeled secondary antibodies (A-B). Nuclei were stained with Hoechst33342, a cell membrane permeable, DNA-binding fluorophor staining nuclei of cells with blue fluorescence.
(TIFF)

Figure S2 **Flow cytometry analysis for detection of CD74 on the cell surface.** Cells were labeled with anti-CD74-FITC (BD Bioscience, Heidelberg,Germany) or the appropriate isotype control. Blue line indicates CD74 and grey shaded area the isotype control.
(TIF)

Figure S3 **Total protein levels remained unchanged after preconditioning.** Representative results from western-blotting analysis were illustrated at distinct time points to demonstrate that total protein levels remained unchanged after preconditioning with 1.5% isoflurane for 4 h by western blotting. Band intensities were normalized to the total kinase levels (unphosphorylated + phosphorylated).
(TIF)
