# Supplementary figures and images for: The Role of Macrophage Migration Inhibitory Factor in Anesthetic-Induced Myocardial Preconditioning
Source: PLoS One. 2014 Mar 25;9(3):e92827. doi: 10.1371/journal.pone.0092827 (PMC3965449; doi:10.1371/journal.pone.0092827)

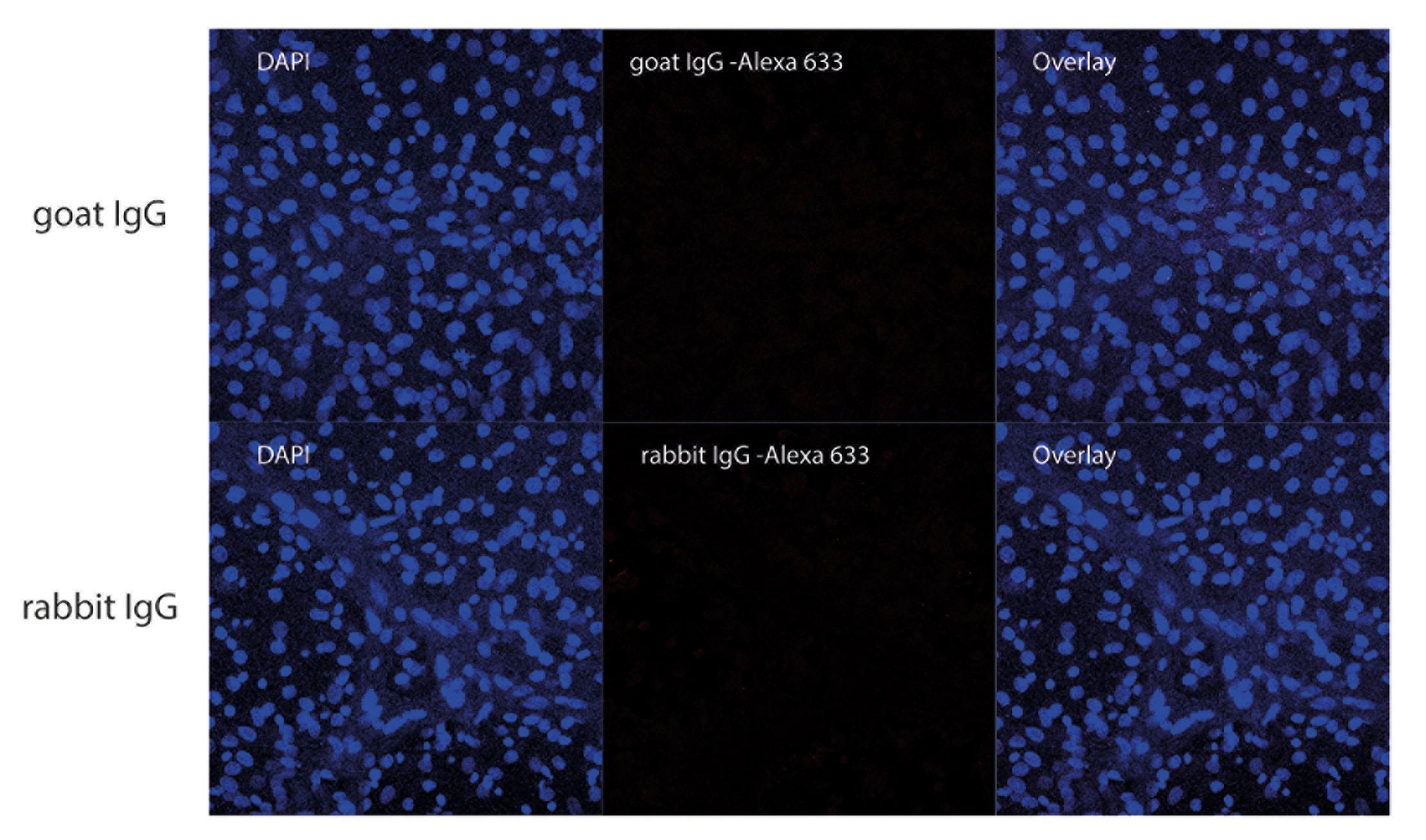

Supplement: Figure S1 — Control - characterization of MIF receptors by confocal microscopy. Rat cardiomyocytes were grown in an Ibidi μ-dish for 12 days. They were fixed with paraformaldehyde, permeabilised with Triton X-100, treated with the appropriate isotype controls and fluorescently labeled secondary antibodies (A-B). Nuclei were stained with Hoechst33342, a cell membrane permeable, DNA-binding fluorophor staining nuclei of cells with blue fluorescence. (TIFF) [file pone.0092827.s001.tif]

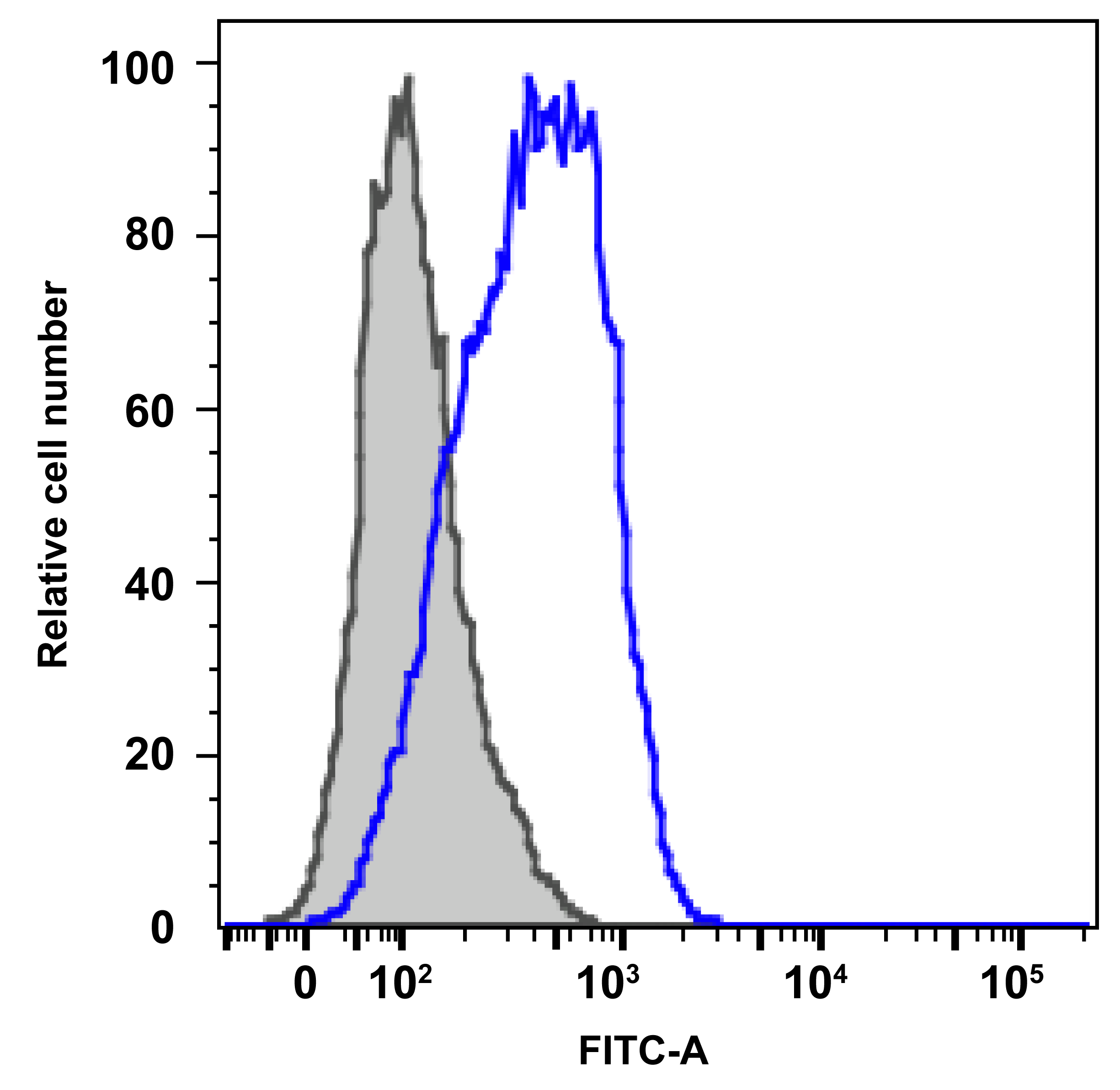

Supplement: Figure S2 — Flow cytometry analysis for detection of CD74 on the cell surface. Cells were labeled with anti-CD74-FITC (BD Bioscience, Heidelberg,Germany) or the appropriate isotype control. Blue line indicates CD74 and grey shaded area the isotype control. (TIF) [file pone.0092827.s002.tif]

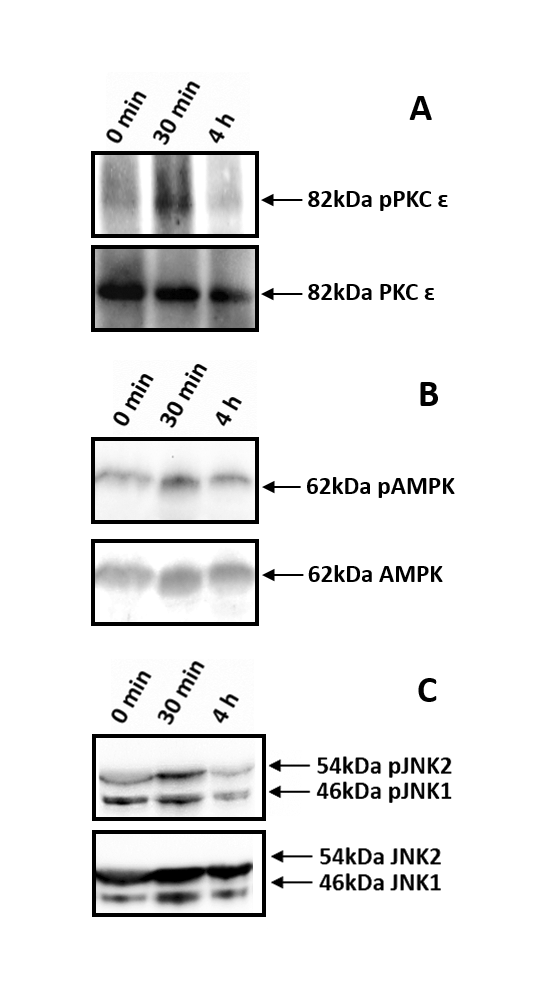

Supplement: Figure S3 — Total protein levels remained unchanged after preconditioning. Representative results from western-blotting analysis were illustrated at distinct time points to demonstrate that total protein levels remained unchanged after preconditioning with 1.5% isoflurane for 4 h by western blotting. Band intensities were normalized to the total kinase levels (unphosphorylated + phosphorylated). (TIF) [file pone.0092827.s003.tif]
